# Effects of exogenous spraying of melatonin on the growth of *Platycrater arguta* under drought stress

**DOI:** 10.3389/fpls.2024.1516302

**Published:** 2025-01-17

**Authors:** Xule Zhang, Xiaohua Ma, Yaping Hu, Qingdi Hu, Junxiu Wen, Yizeng Chen, Renjuan Qian, Jian Zheng

**Affiliations:** Key Laboratory of Plant Innovation and Utilization, Institute of Subtropical Crops of Zhejiang Province, Wenzhou, China

**Keywords:** endangered plants, stress response, physiology and biochemistry, transcriptome, plant hormones

## Abstract

*Platycrater arguta* is a rare and endangered deciduous shrub originating from the Tertiary Period. Understanding its drought tolerance is crucial for conservation efforts and enhancing its resilience to environmental stress. This study aimed to assess the effects of varying levels of drought stress on the phenotype and physiological–biochemical characteristics of *P. arguta*. The study subjected *P. arguta* to different levels of drought stress using 10%, 20%, and 30% polyethylene glycol-6000 (PEG-6000) over a 10-day period. Additionally, the effects of exogenous melatonin application at various concentrations (including 100 µM) were examined to determine its potential in alleviating drought-induced damage. Key parameters measured included leaf relative water content, net photosynthetic rate (Pn), antioxidant enzyme activity, and soluble sugar content. Drought stress significantly inhibited the growth of *P. arguta*. As PEG-6000 concentration increased, leaf relative water content and net photosynthetic rate decreased, while leaf wilting severity, membrane damage, antioxidant enzyme activity, and soluble sugar content increased. A 30% PEG-6000 concentration caused irreversible damage, leading to plant death. Exogenous application of 100 µM melatonin alleviated this damage by increasing leaf relative water content, enhancing photosynthetic efficiency, boosting antioxidant enzyme activity, accumulating osmotic regulators, and reducing leaf desiccation. The study demonstrated that *P. arguta* is sensitive to severe drought conditions, with 30% PEG-6000 causing irreversible damage. However, the application of 100 µM melatonin significantly improved the plant's drought tolerance by upregulating the expression of ABI1 and downregulating genes such as AUX1A-2, IAA2-2, and HP2-1. This finding highlights the potential of melatonin as a protective agent against drought stress, providing valuable insights for the conservation and enhancement of *P. arguta's* resilience to environmental challenges.

## 1 Introduction

*Platycrater arguta* belongs to the genus Platycrater within the family Hydrangeaceae. It is a relict species that existed before Japan separated from the Asian continent and is an endemic, deciduous shrub confined to East Asia (Qi et al., 2014; Manchester et al., 2015; Mallapaty, 2020). Listed as a nationally protected Class II endangered species, it was included in the Red Data Book of Chinese Plants in 1992 (Rizhsky et al., 2002). Its unique floral morphology, aesthetic appeal, high tolerance to pruning, ease of shaping, and rapid propagation make it widely used in flowerbeds and indoor potted displays, contributing significantly to both ornamental and economic value. Additionally, its leaves can be used as a tea substitute, offering health benefits such as vitality enhancement and vision improvement, indicating broad development potential (Manchester et al., 2015; Wei et al., 2024).

In recent years, drastic global climate changes have brought ecological challenges to the forefront, with frequent occurrences of extreme weather events, natural disasters, and freshwater scarcity. The survival and continuity of endangered species face unprecedented threats, constrained by both biotic and abiotic stresses. Among the abiotic stresses, drought is one of the most common and critical environmental factors affecting plant growth and development (Ahluwalia et al., 2021; Seleiman et al., 2021). Drought stress impacts photosynthetic efficiency (Ghotbi-Ravandi et al., 2014; Zahra et al., 2023), reactive oxygen species (ROS) metabolism (Noctor et al., 2014; Roy et al., 2021), and osmotic regulation (Khan et al., 2021; Ozturk et al., 2021), leading to metabolic imbalances, inhibited growth, and reduced productivity. It not only impairs individual plant survival and development but also restricts population distribution and persistence, posing particularly severe risks to endangered species (Vander Vorste et al., 2020; Rodríguez-Caro et al., 2021).

Melatonin (MT), also known as pineal hormone, can be synthesized endogenously by plants or absorbed from the external environment and accumulated internally. As an indoleamine, MT functions as a vital plant growth regulator (Kvetnoy et al., 2022; Mukherjee et al., 2024). It acts directly to scavenge free radicals and indirectly as an antioxidant, modulating various metabolic pathways to enhance plant stress tolerance. MT has been shown to mitigate the adverse effects of environmental stressors such as heavy metal toxicity (Altaf et al., 2023; Yang et al., 2023), drought (Li et al., 2021, 2022b), low temperatures (Zhang et al., 2021; Li et al., 2022a), and high temperatures (Jia et al., 2020; Raza et al., 2022), thus playing a crucial role in plant adaptation to challenging conditions (Song et al., 2024).

This study investigates the effects of exogenous MT application on the growth of P. arguta under drought stress conditions. P. arguta seedlings were subjected to drought stress simulated by different concentrations (10%, 20%, and 30%) of polyethylene glycol-6000 (PEG-6000) to analyze the phenotypic and physiological responses under varying drought intensities. Subsequently, at the highest stress level (30% PEG-6000), exogenous MT was applied at three concentrations (50 μmol/L, 100 μmol/L, and 200 μmol/L) to assess its regulatory effects on drought-stressed seedlings.

Currently, research on the response of *P. arguta* to drought stress is limited, especially regarding its physiological, biochemical, and molecular mechanisms. This study provides crucial theoretical insights into the growth and adaptation of this endangered species under stress, addressing the gaps in understanding its physiological responses, metabolic changes, and molecular mechanisms in the face of drought. Additionally, while MT, a plant growth regulator, has not been extensively studied for its role in drought stress response, its application in *P. arguta* offers new possibilities for enhancing drought tolerance. This research not only unveils the mechanisms by which MT influences plant stress responses but also highlights its potential as a growth regulator for improving the resilience of endangered plants, offering valuable insights for their conservation and cultivation.

## 2 Materials and methods

### 2.1 Study site and experimental materials

The experiment was conducted at the Germplasm Resource Garden for Regional Ornamental Plants of the Zhejiang Subtropical Crop Research Institute, located in Wenzhou, Zhejiang, China (28°00’47’’ N, 120°64’20’’ E). Two-year-old healthy and robust *P. arguta* seedlings grown from seeds were used as the experimental materials. Six concentration gradients of PEG-6000 (5%, 10%, 15%, 20%, 25%, and 30%) were tested, with 100 ml of the corresponding aqueous solutions applied to each pot daily for 10 days. The test seedlings were placed in a plastic frame to prevent solution leakage and to ensure complete absorption of the PEG-6000 solution. After the treatment period, the relative water content (RWC) and relative electrical conductivity (REC) of the leaves were measured. Based on the results, 10%, 20%, and 30% PEG-6000 solutions were selected to represent mild, moderate, and severe drought stress, respectively, for the main experiment.

### 2.2 Experimental design and treatment application

The formal experiment began in May 2024, using uniformly growing, disease-free 2-year-old *P. arguta* seedlings in pots. To ensure experimental consistency and avoid external environmental interference, all plants were maintained under stable conditions, with a daytime temperature of 25°C and a nighttime temperature of 18°C. No additional treatments were applied during the experimental period.

PEG-6000 solutions were prepared at concentrations of 0%, 10%, 20%, and 30%, and 100 ml of each solution was applied daily to the corresponding treatment groups. Prior to the experiment, the seedlings were thoroughly watered for three consecutive days, with no further irrigation or treatments during the experiment. While treating with PEG-6000 solution, MT was sprayed at around 18:00 in the evening using a fine droplet sprayer. When spraying, pay attention to spraying evenly on the upper and lower surfaces of the leaves. Spraying was continued for 10 days, with the leaf surface and back being wet and not dripping. Normal water and fertilizer management was performed during the spraying period. Sampling was performed between 8:00 and 9:00 in the morning after the treatment. The leaves obtained needed to be quickly frozen in liquid nitrogen and stored in a −80°C refrigerator.

### 2.3 Determination of experimental indicators

#### 2.3.1 Classification of leaf wilting degree

The leaves were mainly observed from several dimensions, including growth potential, leaf color, leaf wilting degree, leaf wilting amount, leaf water loss, and whether the leaves were drooping. The specific situation was shown in **Table 1**.

**Table 1:** Classification of leaf phenotypic observations.

Wilting grade	Leaf symptoms
I	Good growth, normal leaf color, no wilting
II	General growth, normal leaf color, leaves at the base begin to lose water and wilt
III	Leaves begin to lose water and curl, leaves droop, leaf color is basically normal, leaf wilting amount < 20%
IV	Leaves lose water seriously, petioles become soft, leaf wilting amount accounts for 20%–50%
V	Leaves curl and droop seriously, leaf wilting amount accounts for 50%–80%
VI	All leaves wilt

#### 2.3.2 Conductivity measurement

The collected leaves of *P. arguta* were rinsed thoroughly with deionized water, promptly patted dry, and the midribs were removed. The leaves were then cut into small pieces of 2 cm × 2 cm, thoroughly mixed, and accurately weighed to obtain 0.08 g, which was subsequently placed into a test tube. Deionized water was added to the test tube in a ratio of leaf fresh weight (g) to deionized water (ml) of 1:9. The test tube was gently shaken. After allowing the mixture to stand at room temperature for 24 hr, the initial conductivity (C1) was measured using a conductivity meter. Following the initial measurement, the test tube was placed in a high-temperature water bath and boiled for 30 min, then cooled to room temperature before measuring the final conductivity (C2) (Konsta et al., 1997).


Conductivity(%)=C1/C2×100%

#### 2.3.3 Measurement of leaf relative water content

The oven-drying method to constant weight was utilized. Initially, the leaves of *P. arguta* were weighed to obtain their fresh weight (Wf) using a balance and then promptly placed into envelopes. Following the weighing, the plant leaves, along with the envelopes, were placed into an oven preheated to 105°C for 15 min to inactivate enzymes (blanching). Subsequently, they were transferred to an oven maintained at 80°C and dried until a constant weight was achieved, at which point the dry weight (Wd) was measured.


Leaf Relative Water Content(%)=(Wd/Wf)×100%

#### 2.3.4 Photosynthesis measurement

Photosynthetic gas exchange parameters of *P. arguta* leaves under drought stress were measured using a Li-6400 Portable Photosynthesis System (Li-Cor, USA) with a non-transparent chamber between 9:00 and 11:30 a.m. on sunny days. During each measurement, healthy, intact leaves free from pests and diseases were selected from the plants. The parameters of the photosynthesis system were set as follows: CO2 concentration at 400 μmol·mol^−^¹ and saturated light intensity within the leaf chamber at 1200 μmol·m^−^²·s^−^¹. After the system stabilized, the net photosynthetic rate (Pn), intercellular CO2 concentration (Ci), stomatal conductance (Gs), and transpiration rate (Tr) of the leaves were measured. A total of six replicates were conducted, with 20 observations per replicate, and the average value was taken as the measured value.

The photosynthetic pigment content was determined by direct extraction method (Croft and Chen, 2018).

#### 2.3.5 Determination of physiological indicators

The measurements of MDA, H_2_O_2_, SOD, POD, CAT, SS, and Pro all require the use of crude enzyme extract. The preparation method for crude enzyme extract is as follows: the veins of *P. arguta* leaves were removed and the leaves were cut into small pieces. One gram of the leaf pieces was placed in a pre-cooled mortar and ground into a homogenate under liquid nitrogen. The homogenate was then poured into a test tube, and 9 ml of phosphate buffer solution with a concentration of 0.1 mol·L^−^¹ and a pH of 7 were added. The centrifuge was set to a temperature of 4°C and a speed of 10,000 r/min, and the mixture was centrifuged for 10 min. After the mixture was stratified, the supernatant, which served as the crude enzyme extract, was collected and stored in a refrigerator at 4°C for future use.

The MDA content was measured using the thiobarbituric acid (TBA) colorimetric method (Schmedes and Hølmer, 1989). The SOD content was determined by the nitroblue tetrazolium (NBT) photoreduction method (Cheng et al., 2015). The POD activity was assayed using the guaiacol colorimetric method (Doerge et al., 1997). The CAT activity was measured by the ultraviolet absorption method (Mach et al., 1995). The SS content was determined by the anthrone colorimetric method (Leng et al., 2016). The Pro content was assayed using the acidic ninhydrin colorimetric method (Doi et al., 1981).

## 3 Results

### 3.1 Effects of drought stress on the phenotype of *P. arguta*

**Figure 1:**
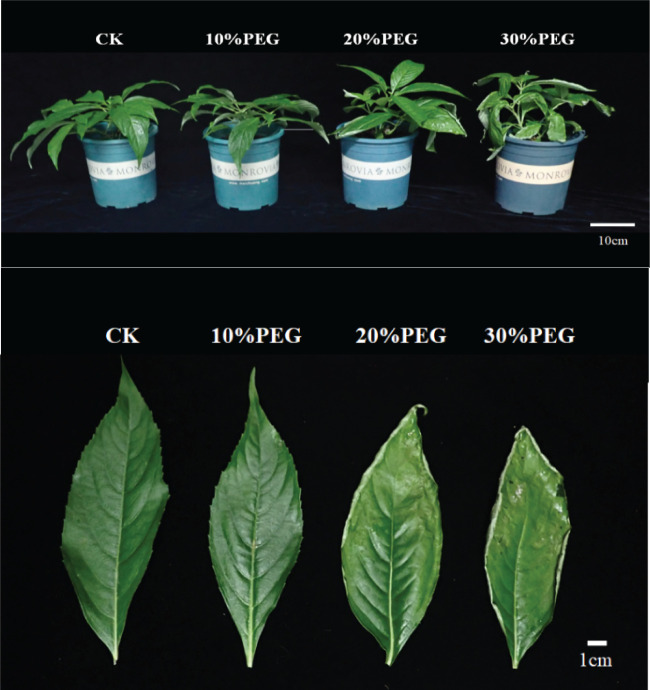
Effect of PEG-6000 on the phenotype of *P. arguta* after 10 days treatment.

Under control conditions (CKs), *P. arguta* plants exhibited vigorous growth with normal green leaves, classified as Grade I. Compared to CK, plants subjected to different concentrations of PEG-6000 showed varying growth responses. At 10% PEG-6000, no significant changes were observed in leaf morphology, maintaining Grade I status. At 20% PEG-6000, slight leaf curling occurred without wilting or drooping, classified as Grade IV. At 30% PEG-6000, leaves exhibited severe wilting, desiccation, and even abscission, indicating significant water loss and classified as Grade VI (**Figure 1**).

**Figure 2:**
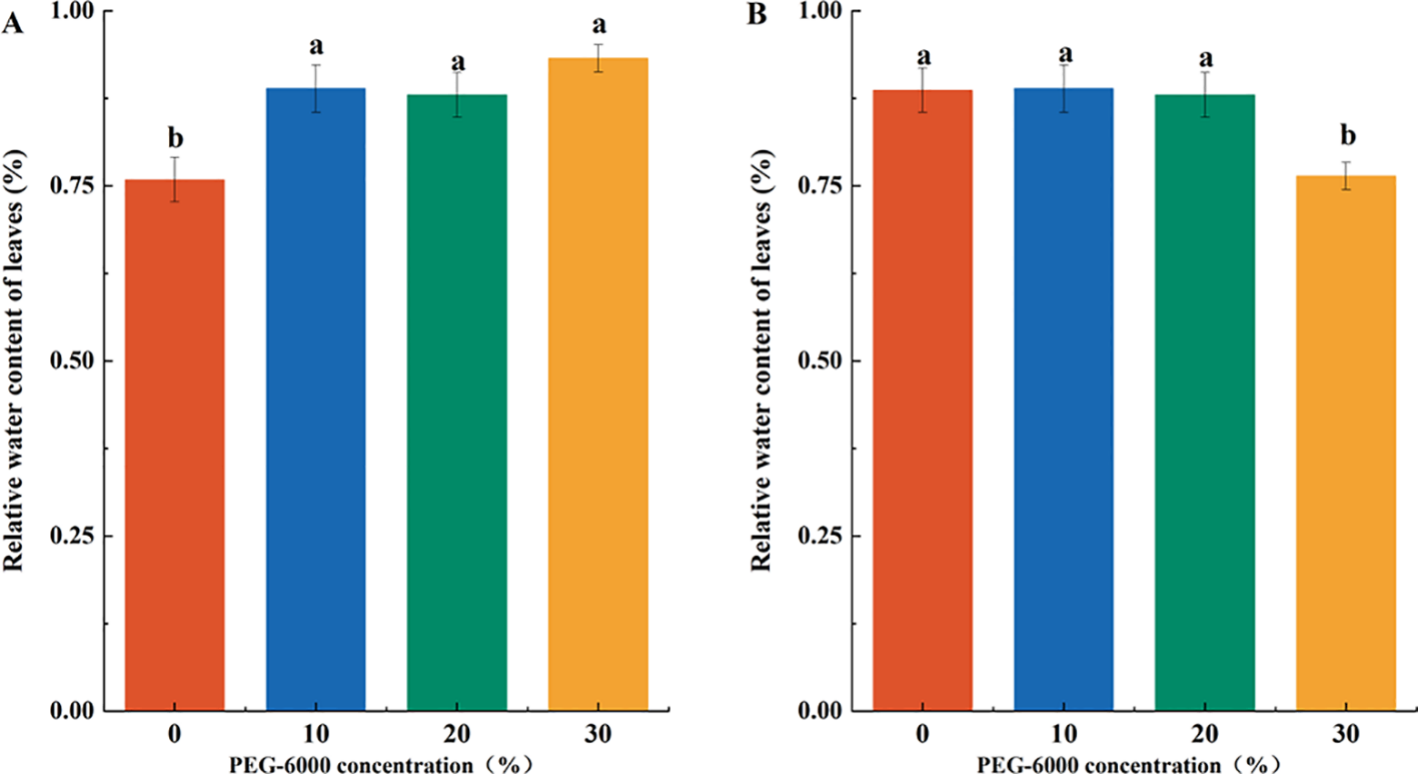
Effects of PEG-6000 on REC **(A)**, RWC **(B)** of *P. arguta* leaves after 10 days treatment. Different letters indicate significant differences (P < 0.05).

Drought stress resulted in leaf wilting, reduced RWC, increased membrane permeability, and enhanced electrolyte leakage. At 10% PEG-6000, RWC showed no significant change compared to CK. At 20% PEG-6000, a declining trend in RWC was observed, though not statistically significant. However, at 30% PEG-6000, the leaves wilted, dried out, and abscised, with a significantly lower RWC-12% less than the CK group (**Figure 2A**). The REC of the leaves increased with PEG-6000 concentration, indicating membrane damage. After 10 days of drought stress, the REC values at 10%, 20%, and 30% PEG-6000 were 1.33, 1.87, and 2.35 times that of the CK group, respectively, with significant differences among treatments (**Figure 2B**).

### 3.2 Effects of drought stress on the photosynthetic system

**Figure 3:**
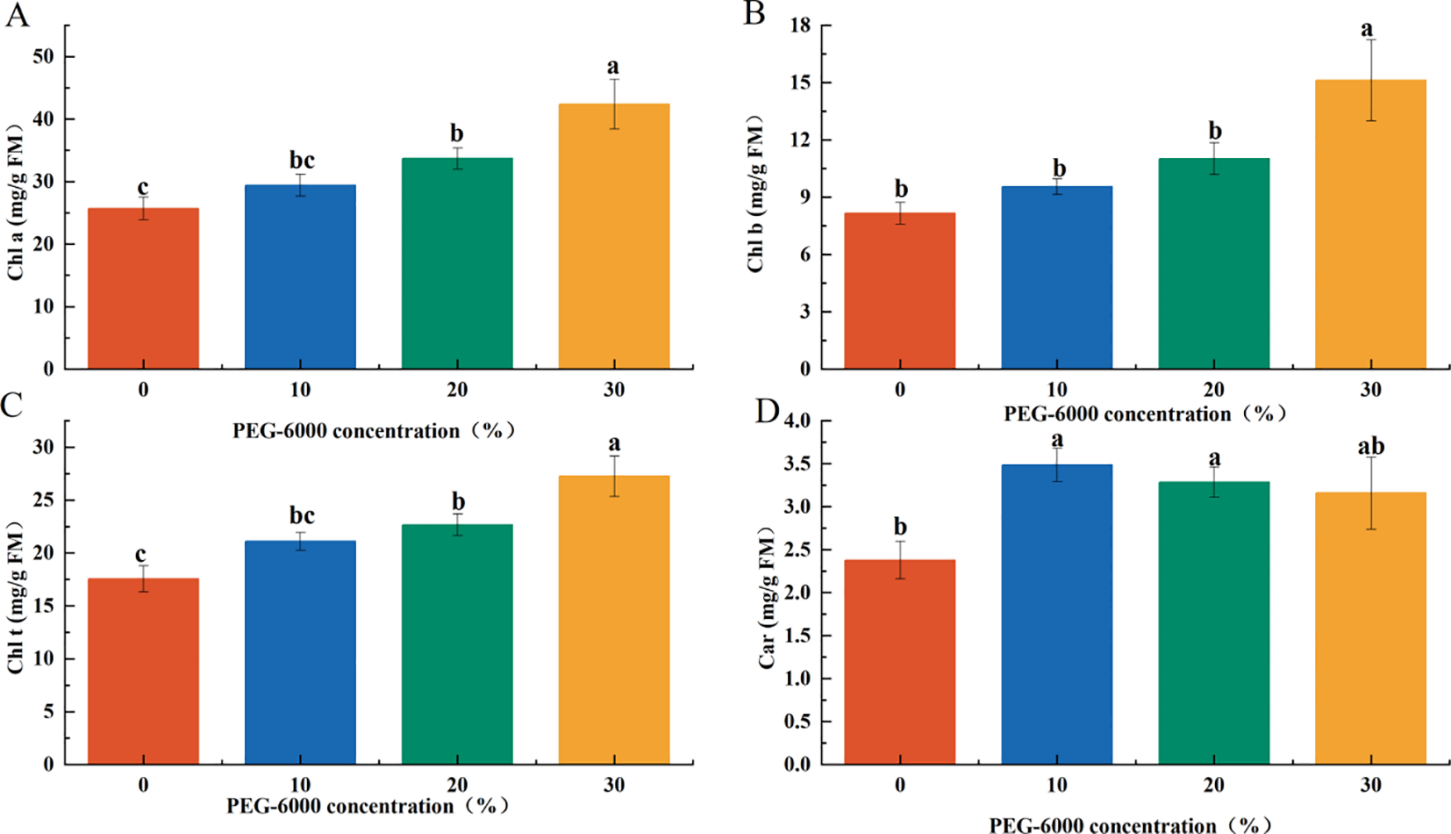
Effects of PEG-6000 on the contents of Chl a **(A)**, Chl b **(B)**, Chl t **(C)**, Car **(D)** in *P. arguta* leaves after 10 days treatment. Different letters indicate significant differences (P < 0.05).

The concentration of photosynthetic pigments increased with PEG-6000 levels, with the 30% PEG-6000 treatment showing significantly higher pigment content than the other groups. Specifically, chlorophyll a (Chl a), chlorophyll b (Chl b), total chlorophyll (Chl t), and carotenoid (Car) contents were 35.59%, 46.06%, 39.32%, and 31.75% higher, respectively, compared to the CK group. The pigment levels in the 10% and 20% PEG-6000 groups were intermediate between those of the CK and 30% PEG-6000 groups (**Figure 3**).

**Figure 4:**
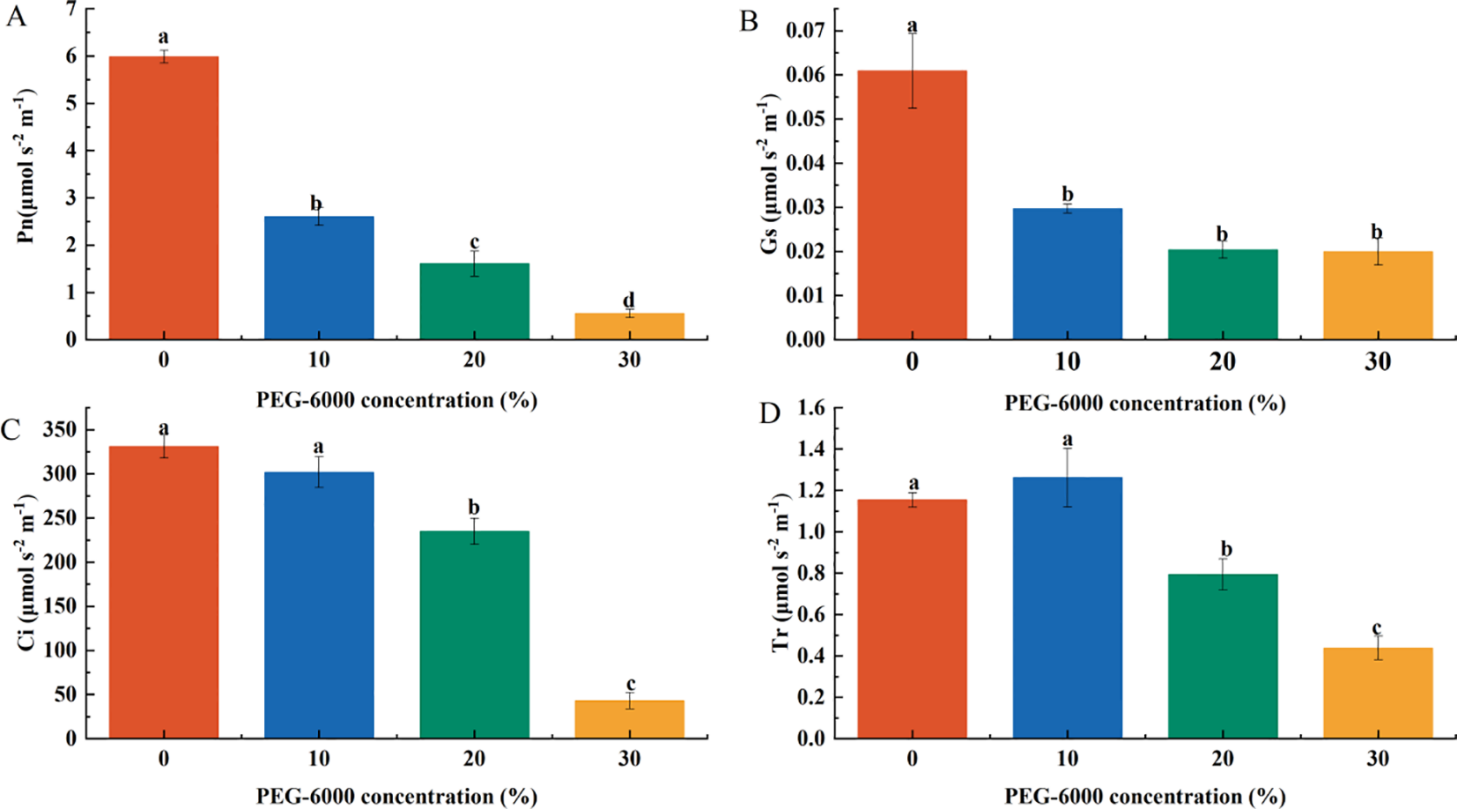
Effects of PEG-6000 on Pn **(A)**, Gs **(B)**, Ci **(C)**, Tr **(D)** of *P. arguta* leaves after 10 days treatment. Different letters indicate significant differences (P < 0.05).

Photosynthetic gas exchange parameters decreased with increasing PEG-6000 concentration. After 10 days of drought stress, the net photosynthetic rate (Pn) at 10%, 20%, and 30% PEG-6000 was 43.52%, 26.83%, and 9.25% of the CK group, respectively, with significant differences among treatments (**Figure 4A**). Stomatal conductance (Gs) also decreased significantly, corresponding to 48.77%, 33.50%, and 32.84% of CK, with no significant differences among the three drought treatments (**Figure 4B**). The intercellular CO2 concentration (Ci) in the 20% and 30% PEG-6000 treatments was significantly lower than that in the CK and 10% PEG-6000 groups, corresponding to 70.96% and 12.92% of CK, respectively, while no significant difference was observed between CK and 10% PEG-6000 (**Figure 4C**). Similarly, the transpiration rate (Tr) was significantly reduced in the 20% and 30% PEG-6000 groups, reaching 68.81% and 37.87% of CK, respectively, with no significant difference between the CK and 10% PEG-6000 groups (**Figure 4D**).

### 3.3 Effects of drought stress on hydrogen peroxide (H_2_O_2_) content

**Figure 5:**
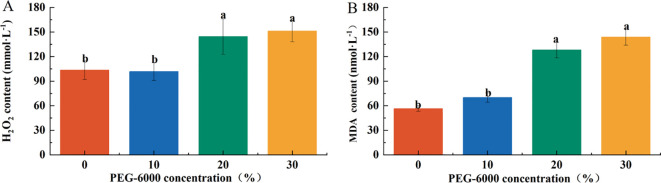
Effects of PEG-6000 on the content of H_2_O_2_ **(A)**, MDA **(B)** *P. arguta* after 10 days treatment. Different letters indicate significant differences (P < 0.05).

The H_2_O_2_ content in leaves increased significantly in the 20% and 30% PEG-6000 treatments, reaching 1.35 and 1.42 times that of CK, respectively. There were no significant differences in H_2_O_2_ content between the CK and 10% PEG-6000 groups nor between the 20% and 30% PEG-6000 groups (**Figure 5A**).

Similarly, the malondialdehyde (MDA) content was significantly higher in the 20% and 30% PEG-6000 treatments, corresponding to 2.27 and 2.55 times that of the CK group, respectively. No significant differences were observed between the CK and 10% PEG-6000 groups or between the 20% and 30% PEG-6000 groups (**Figure 5B**).

### 3.4 Effects of drought stress on antioxidant enzyme activity

The activities of antioxidant enzymes in *P. arguta* leaves exhibited different trends under varying drought stress levels. Superoxide dismutase (SOD) activity decreased with increasing PEG-6000 concentration, while peroxidase (POD) activity increased.

**Figure 6:**
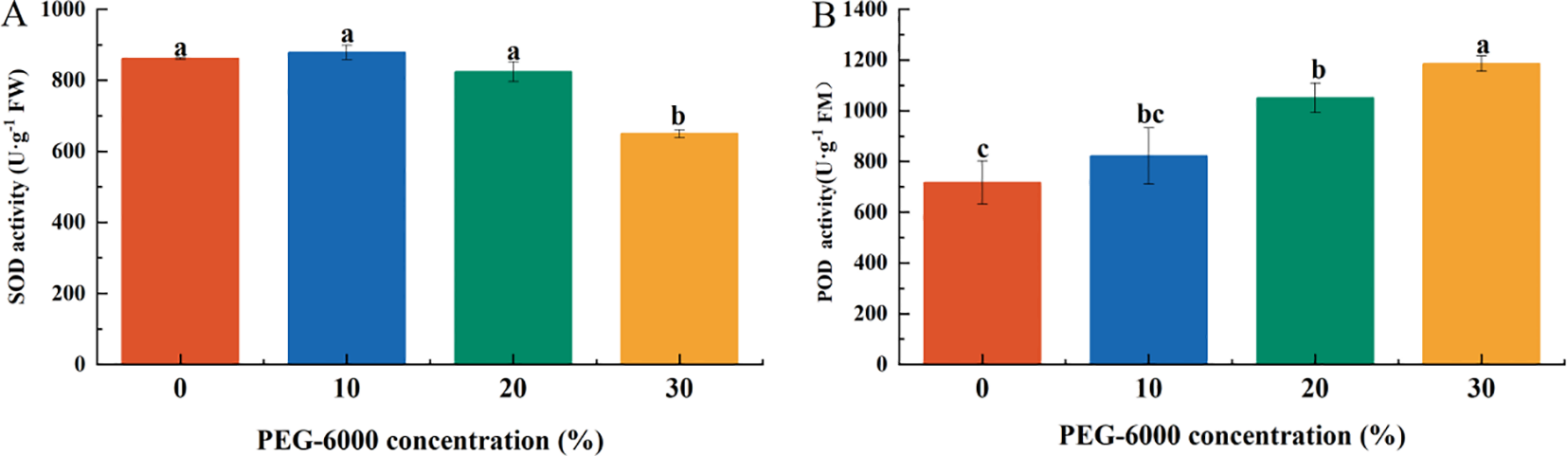
Effects of PEG-6000 on the activities of SOD **(A)**, POD **(B)** *P. arguta* leaves after 10 days treatment. Different letters indicate significant differences (P < 0.05).

After 10 days of drought stress, SOD activity in the 30% PEG-6000 treatment decreased to 75.36% of the CK level, with no significant differences among the other treatments (**Figure 6A**). POD activity in the 20% and 30% PEG-6000 treatments was significantly higher than that in the CK and 10% PEG-6000 groups, corresponding to 1.46 and 1.65 times that of CK, respectively, with no significant differences between the CK and 10% PEG-6000 groups (**Figure 6B**).

### 3.5 Effects of drought stress on osmotic regulatory substances

**Figure 7:**
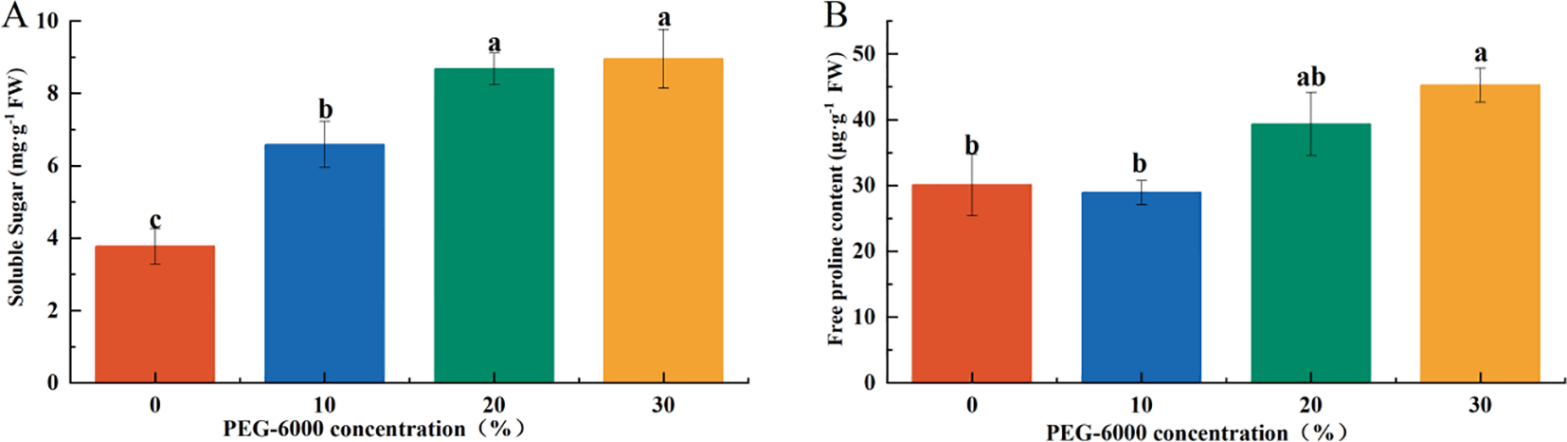
Effects of PEG-6000 on SS **(A)**, Pro **(B)** of *P. arguta* after 10 days. Different letters indicate significant differences (P < 0.05).

The concentration of osmotic regulatory substances increased with the severity of drought stress. Soluble sugar (SS) content in the PEG-6000–treated groups was significantly higher than that of the CK group, corresponding to 1.75, 2.30, and 2.37 times that of CK under 10%, 20%, and 30% PEG-6000, respectively (**Figures 2** **–** **7A**). Under 10% PEG-6000 treatment, proline (Pro) content was significantly lower than that in the 20% and 30% PEG-6000 treatments, with no significant difference compared to CK. The Pro content in the 30% PEG-6000 group was 1.5 times that of the CK group (**Figure 7B**).

### 3.6 Effect of exogenous melatonin on the phenotype of *P. arguta*

**Figure 8:**
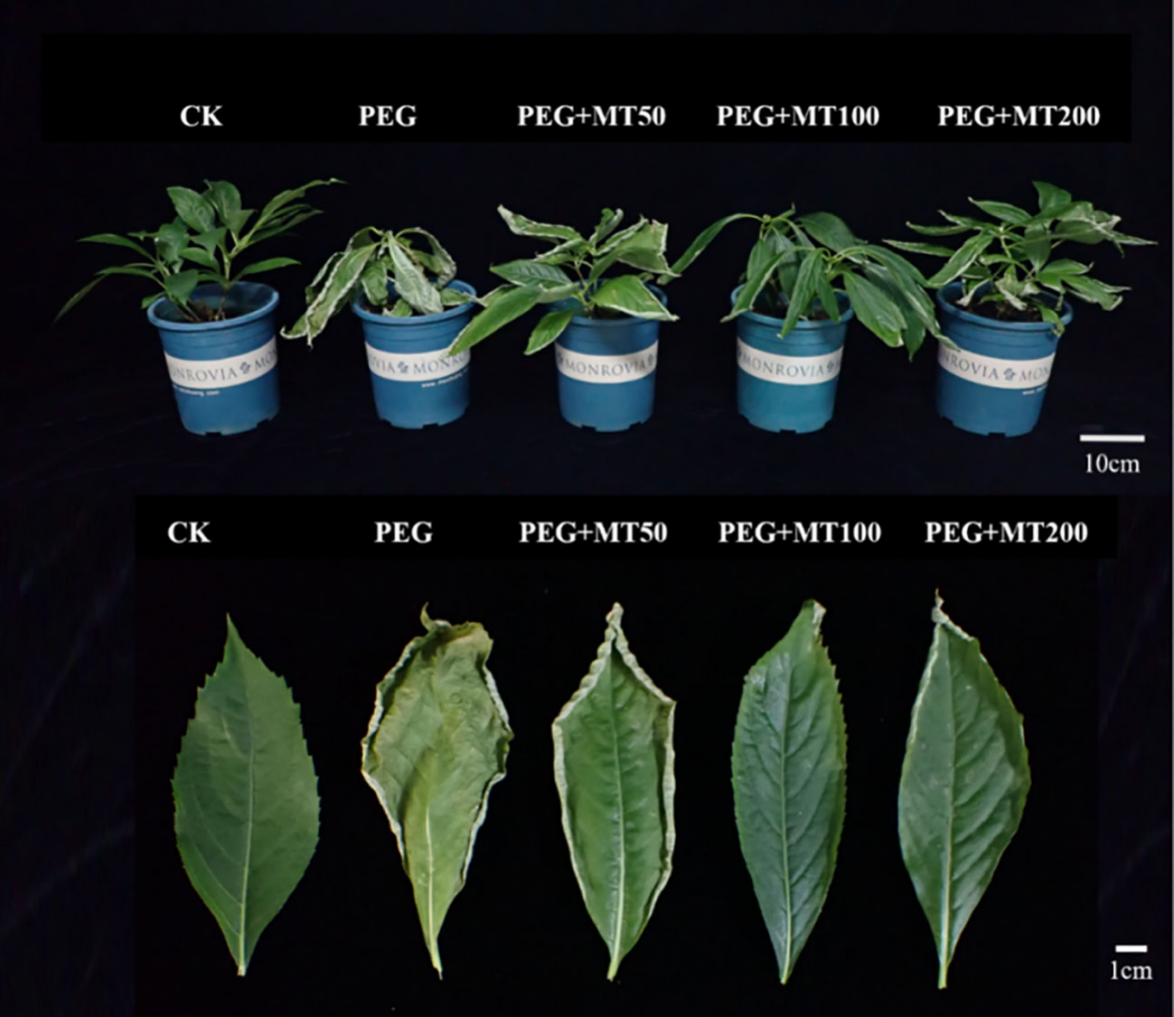
Effects of melatonin on *P. arguta* leaf phenotype under drought stress.

In the control group (CK), leaf color was normal, classified as Grade I. Under treatment with 30% PEG-6000, the leaves of *P. arguta* exhibited severe wilting, reaching Grade VI. As the degree of wilting alleviation varied with different concentrations of MT, the leaves showed distinct growth conditions. Among them, the application of 100 μM MT had the most significant effect, alleviating wilting to Grade III. Treatment with 200 μM was slightly less effective, resulting in Grade IV, while 50 μM had the weakest effect, corresponding to Grade V. Under the 100 μM treatment, the leaf edges curled slightly, but the curled area was minimal. At 200 μM, the leaves exhibited curling without drooping, whereas under the 50 μM treatment, the leaves showed severe wilting, dryness, and even shedding, with pronounced water loss (**Figure 8**).

**Figure 9:**
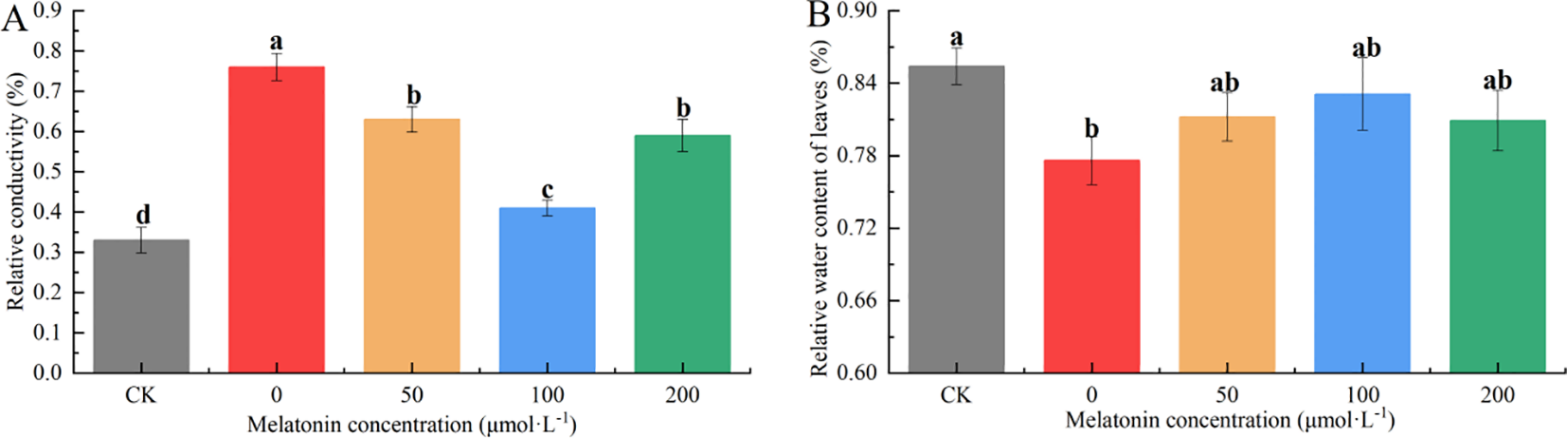
Effects of melatonin spraying on the REC **(A)**, RWC **(B)** of *P. arguta* leaves under drought stress. Different letters indicate significant differences (P < 0.05).

The RWC reflects the extent of water loss in leaves. After 10 days of drought stress, RWC decreased across all treatments. The CK group maintained an RWC of 84.8%, while the group without MT application showed the most significant decline, with an RWC of only 78.6%, significantly lower than the MT-treated groups. Under 50, 100, and 200 μM MT treatments, the RWC values were 81.4%, 83.7%, and 80.4%, respectively, indicating that 100 μM MT most effectively mitigated water loss under drought stress (**Figure 9A**).

The REC reflects the extent of damage to the cell membrane. After 10 days of drought stress, the REC in the group without MT application reached 76.6%, which was 2.55 times that of the CK group. Under 50, 100, and 200 μM MT treatments, the REC values were 2.10, 1.38, and 2.07 times that of the CK group, respectively. These results indicate that MT application can effectively reduce REC, with 100 μM showing the most significant reduction (**Figure 9B**).

### 3.7 Effect of exogenous melatonin on the photosynthetic system of *P. arguta*

**Figure 10:**
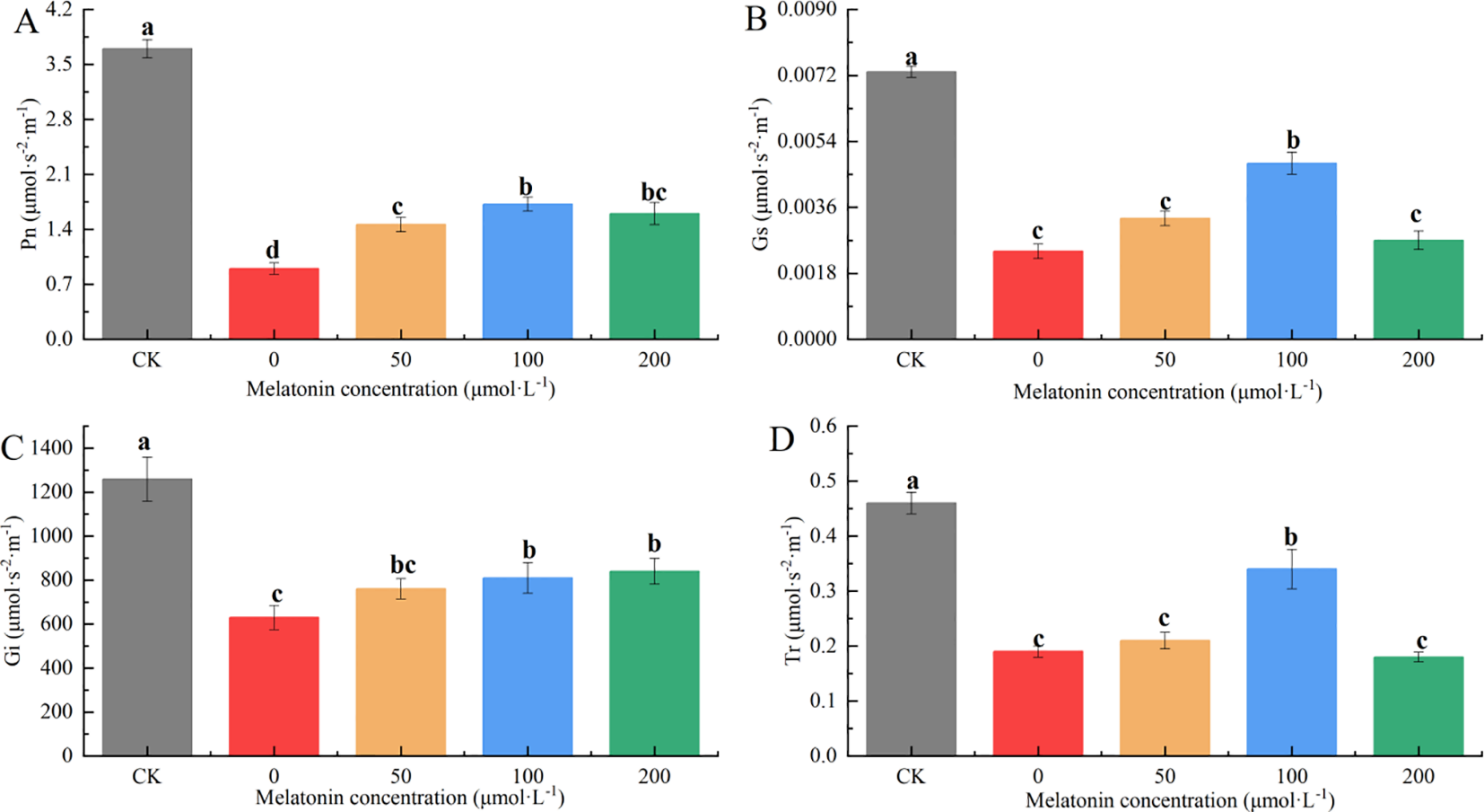
Effects of Melatonin Spraying on the contents of Pn **(A)**, Gs **(B)**, Ci **(C)**, Tr **(D)** of *P. arguta* leaves under drought stress. Different letters indicate significant differences (P < 0.05).

After 10 days of treatment, significant differences in net photosynthetic rate (Pn) were observed among the groups. The Pn values for the other treatment groups were 22.5%, 37.5%, 46.0%, and 41.7% of the CK group, respectively. Among them, the application of 100 μM MT most effectively mitigated the impact of drought stress on photosynthesis (**Figure 10A**). Significant differences were observed in stomatal conductance (Gs) between the CK group, the 100 μM treatment group, and the other treatments. The CK group had the highest Gs value, followed by the 100 μM group. The Gs values in the other groups were 62.16%, 55.41%, 37.84%, and 60.81% lower than those of the CK group, respectively (**Figure 10B**). The intercellular CO2 concentration (Ci) in the CK group was significantly higher than that in other groups, and the Ci under PEG treatment was notably lower than that of the other treatments. No significant differences in Ci were observed among the different MT concentrations. The Ci values in the other groups were 47.08%, 36.98%, 33.12%, and 31.52% lower than those of the CK group, respectively (**Figure 10C**). Significant differences in transpiration rate (Tr) were also observed between the CK group, the 100 μM treatment group, and the other treatments. The CK group had the highest Tr value, followed by the 100 μM group, with no significant differences among the other treatments. The Tr values in the other groups were 57.90%, 54.05%, 32.34%, and 58.70% lower than those of the CK group, respectively (**Figure 10D**).

Overall, under 30% PEG-6000 treatment, the photosynthetic efficiency of *P. arguta* decreased to varying degrees. MT application effectively mitigated the damage caused by drought stress, improving photosynthetic performance, with 100 μM MT showing the most significant alleviating effect.

### 3.8 Effect of exogenous melatonin on the Peroxide content of *P. arguta*

**Figure 11:**
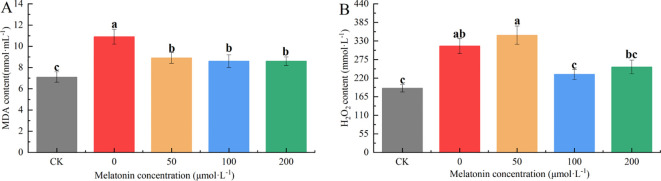
Effects of melatonin on H_2_O_2_ **(A)**, MDA **(B)** contents in *P. arguta* leaves under drought stress. Different letters indicate significant differences (P < 0.05).

The MDA content in the CK group was significantly lower than that of the other groups. The MDA levels in the other groups were 1.63, 1.33, 1.28, and 1.28 times that of the CK group, respectively. The PEG-treated group exhibited significantly higher MDA levels than the MT-treated groups, with no significant differences among the different MT concentrations (**Figure 11A**). The hydrogen peroxide (H_2_O_2_) content in the CK and 100 μM treatment groups was significantly lower than in the other groups, followed by the 200 μM group. The PEG and 50 μM groups had the highest H_2_O_2_ levels, significantly higher than the other treatments. The H_2_O_2_ content in the other groups was 83.78%, 97.21%, 27.84%, and 41.35% higher than that of the CK group, with the 50 μM group reaching nearly double that of the CK group, indicating more severe stress (**Figure 11B**).

### 3.9 Effect of exogenous melatonin on the activity of antioxidant enzymes in *P. arguta*

**Figure 12:**
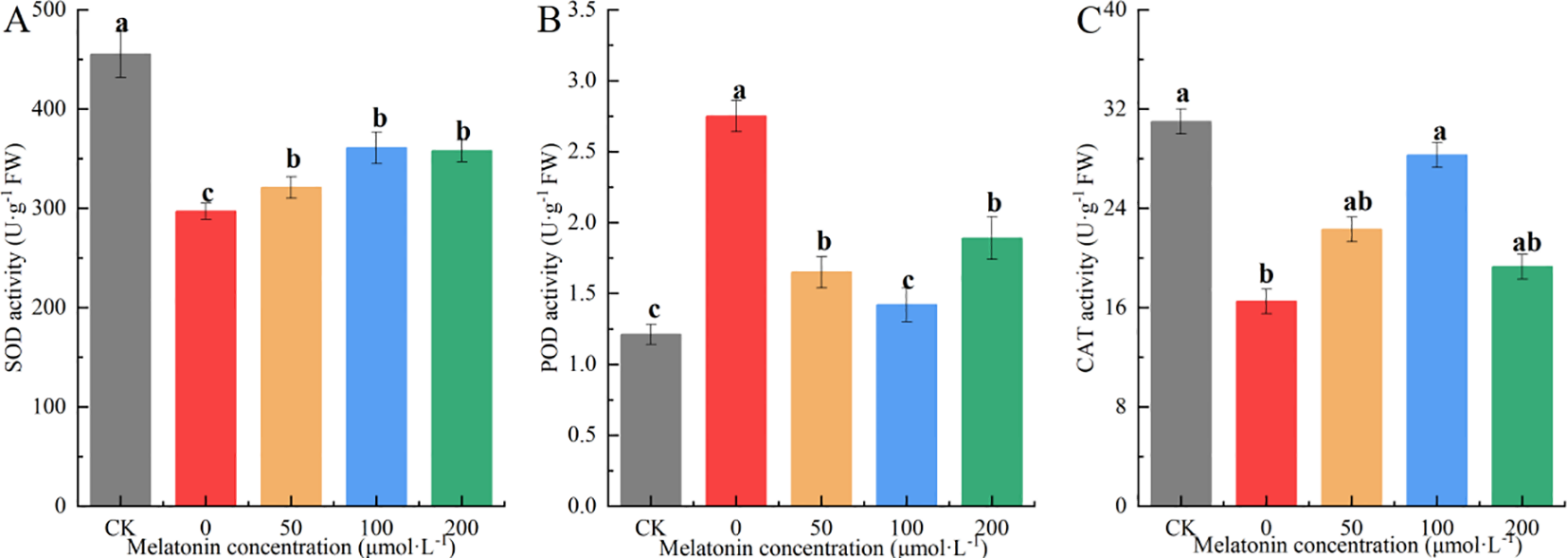
Effects of melatonin on SOD **(A)**, POD **(B)**, CAT **(C)** contents in *P. arguta* leaves under drought stress. Different letters indicate significant differences (P < 0.05).

Variance analysis showed that after 10 days of drought stress, the SOD activity in the drought-stressed group was significantly lower than in other groups. The SOD activity in the MT-treated groups was between that of the CK and drought-stressed groups, with no significant differences among different MT concentrations. The SOD activity in the other groups was 51.97%, 10.13%, 26.76%, and 25.61% higher than that of the drought-stressed group, respectively (**Figure 12A**).

The trend in POD activity differed from that of SOD. The 100 μM MT treatment resulted in POD activity similar to the CK group, which was significantly lower than that of the other groups. The 50 μM and 100 μM treatments showed intermediate POD activity levels, while the PEG-treated group exhibited the highest POD activity, 2.27 times that of the CK group (**Figure 12B**). CAT activity decreased under drought stress, but the 100 μM MT treatment effectively mitigated this decline, bringing CAT activity to a level comparable with the CK group. CAT activity in the 50 μM and 200 μM treatment groups was 76.25%, 38.42%, 69.75%, and 19.95% higher than that of the drought-stressed group, respectively. These results indicate that MT application increases CAT activity under drought stress, with the 100 μM treatment showing the most significant effect (**Figure 12C**).

### 3.10 Effect of exogenous melatonin on osmotic regulators in *P. arguta*

**Figure 13:**
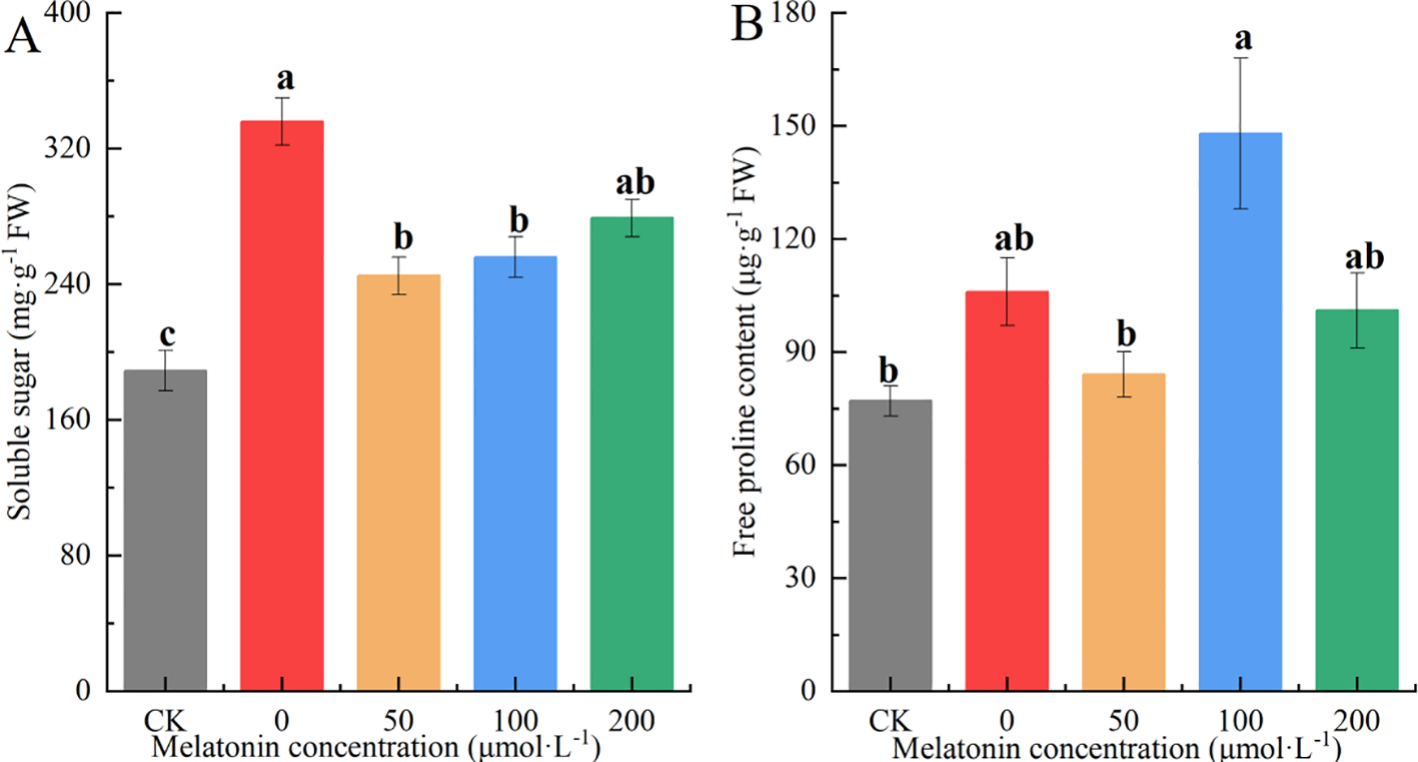
Effects of different concentrations of melatonin on SS **(A)**, Pro **(B)** contents in *P. arguta* leaves under drought stress. Different letters indicate significant differences (P < 0.05).

The SS content in the CK group was significantly lower than in other groups. SS levels in the other groups were 77.12%, 40.92%, 47.25%, and 60.18% higher than those of the CK group, respectively, with the 50 μM group showing the lowest SS content among the MT-treated groups (**Figure 13A**). The proline (Pro) content was highest in the 100 μM treatment group, followed by the PEG and 200 μM groups, with the CK and 50 μM groups having the lowest levels. Pro content in the other groups was 30.38%, 9.86%, 90.14%, and 29.61% higher than that of the CK group, respectively (**Figure 13B**).

### 3.11 Transcriptome sequencing, gene function annotation, and identification of differentially expressed genes

In this experiment, five treatments were selected for transcriptome sequencing: normal water supply (CK), PEG (T1), PEG + MT50 (T2), PEG + MT100 (T3), and PEG + MT200 (T4), with three biological replicates per treatment. A total of 316,538 transcripts were obtained, with a combined length of 301341829 bp. The longest transcript was 17215 bp, and the average length was 952 bp. All transcripts were aligned to six major databases for annotation, and the annotation results were statistically analyzed. Of the unigenes, 37.88% were successfully annotated in all six databases. Comparison with the NR database revealed that the top 5 species most similar to the spiderwort transcriptome were *Selaginella moellendorffii*, *Ceratodon purpureus*, *Marchantia polymorpha*, *Physcomitrium patens*, and *Marchantia paleacea*.

GO annotation of the unigenes indicated that Biological Process annotations were primarily enriched in cellular processes and metabolic processes; Cellular Component annotations were mainly associated with cell parts, cell membrane parts, and organelles; Molecular Function annotations were primarily enriched in binding and catalytic enzyme activities. KEGG pathway analysis showed that the metabolic pathway contained the largest number of genes. A total of 51,644 differentially expressed genes (DEGs) were identified, including 26,053 upregulated and 25,591 downregulated. Among these, 22,070 genes (17.51%) were expressed across all treatments, while 6,725 (5.43%) were specific to CK, 38,384 (30.45%) to PEG, 5,985 (4.75%) to PEG+MT50, 13,383 (10.62%) to PEG+MT100, and 6,061 (4.81%) to PEG+MT200. In pairwise comparisons, PEG+MT100 versus CK revealed 31,870 DEGs (22,324 upregulated, 9,546 downregulated), while PEG+MT100 versus PEG showed 27,068 DEGs (229 upregulated, 26,839 downregulated). Similarly, PEG+MT50 versus PEG+MT100 identified 11,832 DEGs (4,462 upregulated, 7,370 downregulated), and PEG+MT200 versus PEG+MT100 had 22,598 DEGs (5,618 upregulated, 16,980 downregulated).

### 3.12 Differentially expressed gene enrichment analysis

Under drought stress conditions, GO enrichment analysis of DEGs across CK, PEG, and other treatment groups revealed that in the Biological Process category, the enriched pathways were primarily related to cellular processes and metabolic processes. In the Cellular Component category, pathways were mainly enriched in cell parts, organelles, and cell membrane components. In the Molecular Function category, catalytic activity and binding were the dominant enriched pathways. These results indicate that under drought stress, exogenous MT application enhances the drought response of *Sideroxylon dulcificum* by regulating these pathways. Specifically, in the PEG+MT100 versus PEG+MT50 and PEG+MT100 versus PEG+MT200 comparisons, the enriched BP pathways included biosynthetic processes, organic biosynthetic processes, cellular biosynthetic processes, and RNA metabolic processes. Enriched CC pathways involved cellular components, anatomical entities, organelles, and intracellular organelles, while MF pathways were dominated by catalytic activity. This suggests that the PEG+MT100 treatment specifically regulates these pathways, conferring greater drought tolerance at the concentration of 100 μM compared to 50 μM and 200 μM.

KEGG enrichment analysis of DEGs between CK, PEG, and other treatments showed that the primary enriched pathways were related to carbohydrate metabolism, including glycolysis/gluconeogenesis, starch and sucrose metabolism, pentose and glucuronate interconversions, and ribosome-related pathways. These findings suggest that, under drought stress, exogenous MT improves the drought tolerance of *Sideroxylon dulcificum* by modulating carbohydrate metabolism pathways. Additionally, in the PEG+MT100 versus PEG+MT50 and PEG+MT100 versus PEG+MT200 comparisons, differential metabolic pathways included plant hormone signal transduction, POD activity, and carbon fixation in photosynthesis, in addition to the carbohydrate metabolism pathways. This highlights that the PEG+MT100 treatment uniquely regulates these pathways, enhancing drought resistance at 100 μM MT compared to 50 μM and 200 μM concentrations.

### 3.13 Analysis of plant signaling hormone transduction pathways

**Figure 14:**
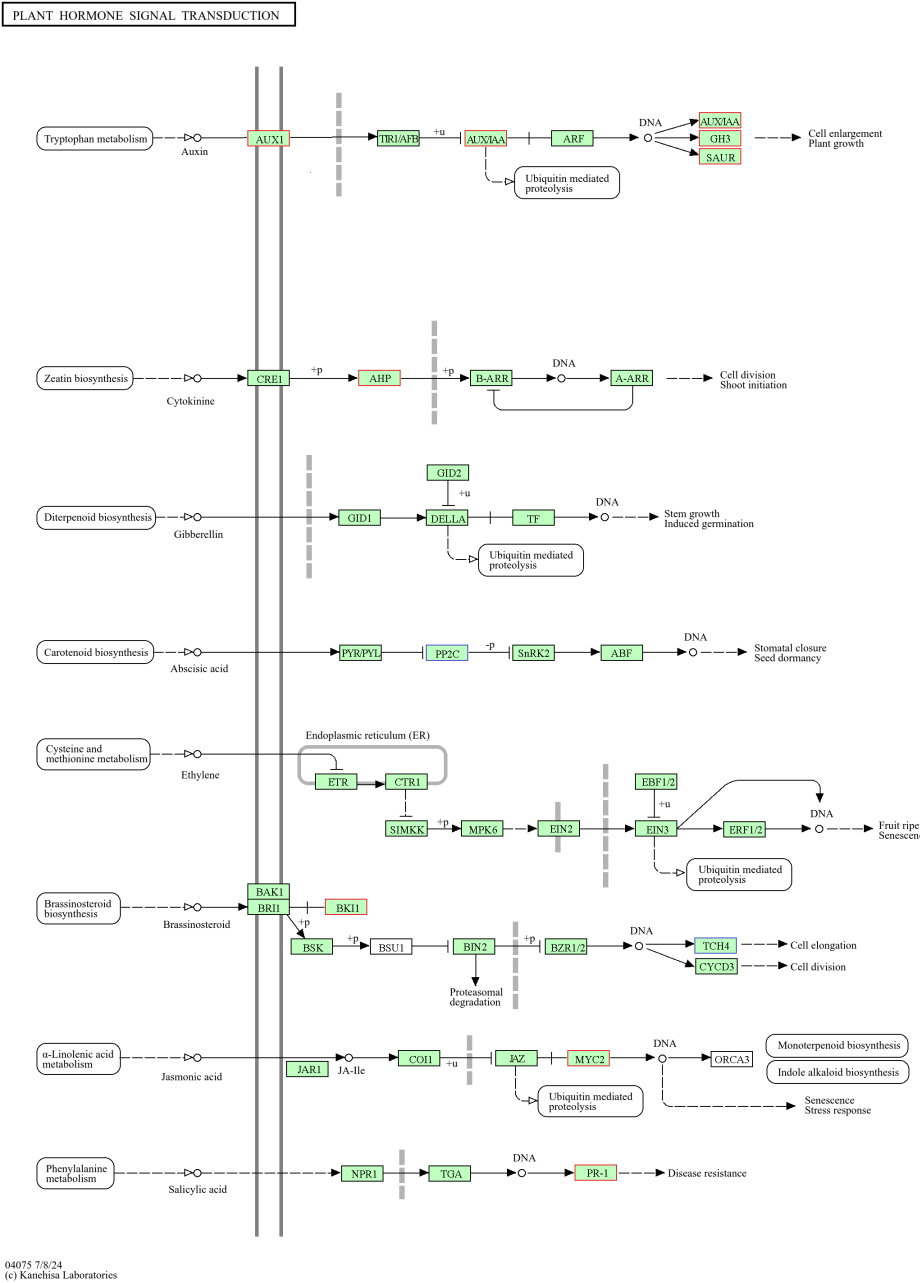
Analysis of plant hormone signal transduction pathways and related differentially expressed genes in PEG+MT50 versus PEG+MT100 combinations. The blue boxes indicate upregulated genes, the red boxes represent downregulated genes. This diagram is based on the KEGG pathway map04075 (Plant Hormone Signal Transduction), modified for clarity.

Among the DEGs related to plant hormone signal transduction in the PEG+MT100 versus PEG+MT50 combination, a total of 16 were identified. Of these, 10 genes associated with the auxin pathway were all downregulated, such as AUX1A-2 (TRINITY_DN877_c0_g1) and IAA2-2 (TRINITY_DN35941_c0_g1). Two genes related to the cytokinin pathway were also downregulated, including HP2-1 (TRINITY_DN9133_c0_g1). In contrast, two genes associated with the abscisic acid (ABA) pathway were upregulated, such as ABI1 (TRINITY_DN3240_c2_g1). Additionally, one gene related to the jasmonic acid pathway and one associated with the salicylic acid pathway were downregulated (**Figure 14**).

## 4 Discussion

The study demonstrates that varying concentrations of PEG-6000 stress inhibit the growth of *P. arguta* and result in a decline in RWC. Among the treatments, the 30% PEG concentration group exhibited the most significant decline, with leaf wilting and shriveling occurring within 4–5 days. After 10 days, leaves dried up, lost water, and even fell off. Drought stress induces water loss in plants, leading to leaf wilting and death, inhibiting plant height, dry matter accumulation, root elongation, and the sprouting of new branches and leaves (Farooq et al., 2009). In this study, as the PEG-6000 concentration increased, RWC showed a decreasing trend to varying degrees, consistent with previous research findings (Altaf et al., 2021).

Drought stress suppresses photosynthesis, with more severe stress resulting in a stronger inhibitory effect (Farooq et al., 2009). Under stress conditions, carotenoids act as antioxidants, scavenging ROS and enabling plants to adapt to adverse environments (Lanza and Dos Reis, 2021). However, studies on the effects of drought stress on chlorophyll content report varying outcomes. For example, Camellia oleifera leaves exhibit a decrease in chlorophyll content with increasing drought intensity and duration (Guo et al., 2023). In Glehnia littoralis, cultivated varieties display an initial decrease in chlorophyll followed by an increase and then a subsequent decline, whereas wild varieties show a continuous decrease, with the lowest chlorophyll concentration observed under 30% PEG-6000 treatment (Lee et al., 2020). In this study, photosynthetic pigment levels increased with stress intensity, possibly due to a reduction in RWC, leading to an increase in dry leaf weight per gram and cell density, which may have caused chlorophyll concentration to rise.

Gas exchange parameters also change under drought stress. Plants under adverse conditions tend to reduce stomatal aperture or close stomata, thereby lowering transpiration rate and stomatal conductance to conserve water, which leads to a decrease in photosynthetic rate (Driesen et al., 2020). In this study, as drought stress intensified, the Pn, Gs, Ci, and Tr of *P. arguta* leaves were all inhibited, supporting these conclusions.

Drought stress initially damages the cell membrane. As the severity of stress increases, membrane permeability rises, resulting in the leakage of more electrolytes and an increase in leaf electrical conductivity. MDA, a key product of lipid peroxidation, reflects the extent of membrane damage (Shao-yu, 1989; Wang YuChao et al., 2010). Studies on Allium species under the same drought conditions found that drought-tolerant varieties exhibit lower REC and higher MDA content (Wu et al., 2009). In this study, treatments with higher PEG-6000 concentrations showed increased leaf electrical conductivity and MDA levels, consistent with previous findings.

Under normal conditions, ROS levels within plant cells remain stable, but drought stress disrupts this balance, leading to lipid peroxidation (Lee et al., 2012; Jajic et al., 2015). Plants have evolved diverse antioxidant mechanisms to detoxify excess ROS and mitigate their harmful effects (Heller and Tudzynski, 2011). Among antioxidant enzymes, SOD plays a dominant role (Alscher et al., 2002). In this study, the 30% PEG-6000 treatment led to increased POD activity, but SOD activity was significantly lower than that under other concentrations. This reduction in SOD activity was accompanied by increased membrane permeability, reduced ROS scavenging capacity, membrane damage, elevated MDA content, and higher RWC. These findings suggest that excessive PEG-6000 concentrations cause irreversible damage to *P. arguta*. Notably, after 10 days of treatment, all leaves under the 30% PEG-6000 condition had fallen off, resulting in plant death.

In response to drought stress, plants actively accumulate osmotic regulatory substances to increase cellular solute concentration, lower osmotic potential, and enhance water retention capacity, helping them cope with adverse conditions (Khaeim et al., 2022). In this study, the contents of SS and free proline in *P. arguta* leaves exhibited an upward trend with increasing PEG-6000 concentration. These results align with earlier studies showing that osmotic regulatory substances increase with rising PEG-6000 concentration and prolonged treatment duration (Qi et al., 2023).

Under the combined influence of drought stress and foliar application of MT at varying concentrations, *P. arguta* exhibited distinct responses in growth, photosynthetic efficiency, antioxidant enzyme activity, and osmoregulatory substances. Specifically, PEG-induced drought stress inhibited the growth of *P. arguta*, with leaf curling, wilting, and a RWC of only around 77%, which was significantly lower than the CK group. MT application alleviated these inhibitory effects, improving plant growth and increasing RWC. Among the tested concentrations, 100 μM MT treatment resulted in the least inhibited growth and the highest RWC. A study on Rosa sertate under 20 days of drought stress similarly found severe leaf wilting, leaf abscission, and stem necrosis, which were mitigated by exogenous MT, with the 200 μM treatment showing the most recovery (Askari-Khorasgani et al., 2021). These findings indicate that MT enhances drought tolerance, though the optimal concentration varies across plant species.

Drought stress reduces photosynthetic efficiency, resulting in a decline in photosynthetic pigments as stress intensifies. Exogenous MT application mitigated this decline, with 50 μM and 100 μM treatments being particularly effective. Previous studies have demonstrated that drought significantly reduces chlorophyll content in Cucumis sativus seedlings, but 100 μM MT treatment increased chlorophyll content by 22.4% compared to drought-stressed plants (Baninasab, 2010). In this study, the photosynthetic efficiency of *P. arguta* declined markedly under drought stress, but MT application effectively alleviated the decline, with the 100 μM concentration yielding the best results. Research has shown that under normal water conditions, MT has minimal impact on wheat photosynthetic parameters, while under drought conditions, MT pre-treatment significantly improves photosynthetic capacity, with 25 μM pre-treatment being the most effective (Zuo et al., 2017).

Drought stress disrupts the integrity of plant cell walls, increasing membrane permeability, as reflected by increased levels of MDA, H_2_O_2_, and REC in this study. MT application reduced the damage to cell membrane permeability, leading to decreased MDA and H_2_O_2_ levels and lower REC values. A study on Zanthoxylum bungeanum reported a significant increase in MDA under drought stress, with MDA levels in the drought-only group being 33% higher than those in the combined drought and MT treatment group (Zhang et al., 2024). Similarly, H_2_O_2_ levels increased dramatically under drought stress, by 2.0- and 1.4-fold compared to the combined treatment, indicating that drought triggers excessive ROS production, which MT effectively mitigates.

Antioxidant enzymes play a critical role in scavenging excess ROS, thereby enhancing drought tolerance (Zheng et al., 2023). In this study, the activities of SOD and CAT were significantly lower in drought-stressed plants compared to the control group, while MT-treated plants showed enhanced SOD and CAT activity. Additionally, POD activity was significantly higher under drought stress than in the control group but decreased significantly with MT application, suggesting that MT regulates antioxidant enzyme activities to better protect *P. arguta* from drought-induced damage. A similar study on Lolium perenne found that SOD and POD activities were significantly lower under drought stress than in the control, but MT treatment notably increased their activity (Wei et al., 2023).

In summary, exogenous application of MT alleviates PEG-induced damage in *P. arguta* by increasing leaf RWC, enhancing photosynthetic efficiency, boosting antioxidant enzyme activity, accumulating osmotic regulatory substances, and reducing leaf desiccation. Moreover, MT improves drought tolerance in *P. arguta* through mechanisms such as promoting the upregulation of indole and its derivatives, reducing the levels of auxins and cytokinins, increasing abscisic acid content, and modulating metabolic pathways related to nicotinic acid and nicotinamide metabolism as well as diterpenoid biosynthesis. The drought adaptability of *P. arguta* is further strengthened by the upregulation of ABI1 and the downregulation of genes such as AUX1A-2, IAA2-2, and HP2-1 under drought stress conditions.

## 5 Conclusions

This study demonstrates that drought stress severely impacts the growth and physiology of *P. arguta*, causing leaf wilting, reduced photosynthesis, and increased oxidative stress. Exogenous MT, particularly at 100 μM, effectively alleviated these effects by enhancing antioxidant enzyme activities, stabilizing membrane integrity, and improving water retention. These findings highlight MT’s potential as a regulatory agent to boost drought tolerance in endangered plants, offering practical insights for conservation and horticultural applications. Further research should focus on long-term field studies and the molecular mechanisms underlying MT’s role in stress mitigation.

## Data Availability

The raw data supporting the conclusions of this article will be made available by the authors, without undue reservation.
